# Beneficence‐Based Obligations and Ethics Consultation in Assisted Dying

**DOI:** 10.1111/bioe.70074

**Published:** 2025-12-26

**Authors:** Georg Marckmann, Anna Hirsch

**Affiliations:** ^1^ Institute of Ethics History and Theory of Medicine Munich Germany

**Keywords:** assisted dying, beneficence, ethics consultation, respect for autonomy, shared decision making, well‐being

## Abstract

In ethical debates on assisted dying, the principle of respect for autonomy is usually invoked to justify respecting requests for assisted dying. However, there are not only autonomy‐based obligations, but also obligations arising from the principle of beneficence towards persons requesting assisted dying. What beneficence requires from persons providing assistance in dying is, however, far less obvious. We will argue that obligations of beneficence, in contrast to autonomy‐based obligations, cannot fulfil a gatekeeping function in regulating access to assisted dying, that is, differentiate between ethically justified and unjustified cases. Nevertheless, they can and should play an important role in this context: Based on philosophical considerations on the concept of well‐being, which is the focus of the principle of beneficence, we elaborate three different roles of beneficence‐based obligations in the context of assisted dying: providing high standards of care, supporting autonomous decision‐making and offering ethical guidance for health care professionals who consider providing assistance in dying. As we will show, these beneficence‐based obligations can also support ethics consultation in assisted dying, especially in cases where there is ethical uncertainty among the assisting persons about whether they should follow the request and provide assistance in dying. Based on a principle‐based approach to integrated ethical decision‐making, we will demonstrate how an elaborated understanding of well‐being and the specified beneficence‐based obligations can contribute to balancing competing ethical obligations and promote ethically sound decisions in requests for assisted dying. The suggested approach can be used as a guidance for ethics consultation in requests for assisted dying.

## 1 Introduction

Assisted dying is often justified with reference to the ethical principle of respect for autonomy [1, 2, 3, 4]. Accordingly, the main obligation in evaluating requests for assisted dying is to ensure that the person has sufficient decision‐making capacity, is well informed about her situation and possible courses of action and is making her decision without undue external influences. However, there are also beneficence‐based obligations towards persons seeking assistance in dying, especially if the wish to die arises within the context of a severe physical or mental illness. Yet, what the principle of beneficence requires from persons providing assistance in dying is less obvious and less discussed—especially because it requires an external judgement that dying is in the best interest of the person [5, 6]. This raises, for example, the difficult question by which substantial normative standard we can determine that a person's situation is so bad that ending her life becomes preferable and what this would mean for the ethical legitimacy of assisted dying. Therefore, we would like to explore what role obligations of beneficence can or should play in evaluating and dealing adequately with requests for assisted dying. Based on this analysis, we will then discuss how the autonomy and beneficence‐based arguments can be applied in a structured way to arrive at ethically well‐justified decisions if the ethical obligations are unclear or conflicting. The suggested approach can be used as a guidance for clinical ethics consultations in requests for assisted dying.

We start with a philosophical analysis of the concept of well‐being, which constitutes the focus of beneficence‐based obligations. In particular, we address the distinction between subjective and objective theories of well‐being and argue for a hybrid understanding of well‐being also in the context of assisted dying. Based on these conceptual clarifications, we first assess whether beneficence‐based obligations can be used as gatekeeping criteria in regulating access to assisted dying, that is, for distinguishing between ethically justified and unjustified cases. We will argue that beneficence‐based considerations are neither suitable nor justified to determine ethical legitimacy in assisted dying and therefore should not be used in regulating access to assisted dying. Gatekeeping can only be justified by the protection of autonomous decisions. Nevertheless, beneficence‐based obligations can play an important role in the process of assisted dying. We will explore three different ways in which the principle of beneficence can provide ethical guidance in caring for persons who consider assisted dying: providing high standards of care, supporting autonomous decision‐making, and offering ethical guidance for health care professionals (HCPs) who consider to provide assistance in dying. Finally, we will discuss how beneficence‐based obligations can be incorporated into a structured approach of principle‐based ethical decision‐making, which can serve as guidance for ethics consultations addressing ethical issues in the context of requests for assisted dying.

## 2 Obligations of Beneficence and Well‐Being: Conceptual Foundations

The principle of beneficence is a well‐established norm in patient care that enshrines the obligation of HCPs to protect and promote the well‐being of their patients. While we acknowledge that not only patients request assistance in dying and not only HCPs offer assistance in dying, we do think that the discussions around beneficence and well‐being in patient care are a helpful starting point for analysing the role of beneficence‐based obligations in assisted dying.

Beneficence is one of the four principles of biomedical ethics, elaborated most prominently by Tom L. Beauchamp and James F. Childress. The two authors understand the ‘principle of beneficence’ as a “general moral obligation to act for the benefit of others” [7], p. 218], defining a ‘beneficent action’ very broadly as ‘all norms, dispositions, and actions with the aim of benefiting or promoting the welfare of other persons’ [7, pp. 217–218]. The crucial question now is: What does it imply to benefit a person and promote her well‐being? Beauchamp and Childress do not give a direct and clear answer to it. However, in philosophy, and now also in medical ethics, there is an ongoing debate about the meaning of well‐being—in the sense of what is *good for* a person (and not what is good in a moral or aesthetic sense, e.g.) [8].

Traditionally, philosophical debates on well‐being distinguish between theories that define well‐being *subjectively*, that is, well‐being depends on the attitudes of the well‐being subject, and those that define well‐being *objectively*, that is, well‐being is independent of personal attitudes [9]. An example of a subjective theory of well‐being is the simple desire‐fulfilment theory, according to which well‐being consists in the fulfilment of personal desires [10]. Objective list theories, on the other hand, are an example of objective theories of well‐being: They identify objects, for example, derived from basic human needs, that contribute to the well‐being of every human being, regardless of personal attitudes and preferences [11].

Both purely objective and purely subjective theories of well‐being have their own insurmountable difficulties, which led to the development of hybrid theories including subjective as well as objective elements [12, 13, 14]. Within this context, Alicia Hall and Valerie Tiberius highlight that the subjectivity of well‐being can be understood more broadly than as the dependence of well‐being on personal desires or preferences, since subjects are not only defined by psychological features but by other features as well, such as personal characteristics and life circumstances. Accordingly, objective theories of well‐being can also pay attention to these features and to the *good‐for‐*relation of well‐being. Hall and Tiberius refer to this broader perspective on the subjectivity of well‐being as ‘subject‐dependence’ or ‘subject‐relativity’ [15].

A hybrid approach to well‐being seems also attractive in the medical context since it is characterised by a tension: on the one hand, medicine, by virtue of its function, is legitimately dedicated to a specific area of human well‐being, namely health‐related well‐being. Further, HCPs rely on certain indicators to determine what it means to act in the best interest of their patients. They have to make treatment recommendations and decide about the medical utility or futility of therapies. Sometimes, they don't know anything about their patients' preferences, for example, if patients lack decision‐making capacity and no treatment preferences are known. This suggests that medicine requires a certain objective basis for what constitutes well‐being which could be spelled out, for example, in the form of an objective‐list theory of well‐being. On the other hand, the individuality of each patient must be respected and individual needs and circumstances considered, which speaks in favour of relating objective well‐being goods to the subject of well‐being, that is, the patient, thereby conceptualizing well‐being as objective *and* subject‐relative at the same time. Thus, the result would be a hybrid theory of well‐being consisting of objective goods whose value must then be determined in relation to the well‐being subject, her individual characteristics and circumstances. But how can the objective basis of well‐being be elaborated within the context of assisted dying?

Well‐being goods appreciated by almost every human being are usually related to fundamental abilities and needs. In this context, Dan Brock speaks of ‘primary functions’: ‘human functions that are necessary for, or at least valuable in, the pursuit of nearly all relatively full and complete human life plans’ [16, p. 127]. Examples are well‐functioning organs, capacities to communicate, ambulation, reasoning, and emotional capabilities [16, p. 127]. If primary functions are endangered, there are usually strong reasons from a beneficence perspective to take all necessary actions to avert the danger—due to the great importance of these goods for any life plan, indeed for the ability to act in general. They usually increase the likelihood to achieve well‐being in different areas of life. This applies at least when patients have a good chance of recovery, are generally healthy, and so forth. On the other hand, if a patient is severely limited in her primary functions or no longer possesses some of them (due to illness or aging), HCPs may recognise, also from their external perspective, that her well‐being may be substantially restricted. But can they also determine, based on certain objective, observable criteria, when a person's life is so bad that it would be better for her to die? And can it ever be consistent with the principle of beneficence to help others to die? There is a lively debate in philosophy about whether it can ever be good for a person to choose death over life [6, 17, 18, 19, 20, 21, 22, 23], which we cannot go into any details here. However, clinical experience shows that people in some situations do arrive at the decision that their life is so bad that they prefer to die rather than live on. Patients who are severely restricted in their daily activities and/or suffer from severe symptoms do often refuse life‐sustaining treatments when they feel that their own quality of life is no longer tolerable [17]. Persons also balance continue living versus dying in advance care planning when they plan future care and define limits for life‐sustaining treatment, usually in the event of severe and irreversible brain damage accompanied by significant restrictions on social interaction [24].

From an external perspective, HCPs can recognise, based on certain objective, observable criteria (limitations of primary (health‐related) functions, high symptom burden, short life expectancy, etc.), that a patient's well‐being is significantly impaired. This allows them to assess the relative weight of beneficence‐based obligations to provide life‐sustaining treatment options: the more restricted the patient's primary functions and the more burdensome the symptoms are, the weaker these obligations become. However, only the patient herself can decide when these limitations and burdens are so severe that dying is preferable because we do not have generally accepted standards for this judgement in pluralistic societies. Rather, the individual's own experiences and ideas of a good life play the decisive role in determining the critical ‘tipping point’ at which it becomes better to die than to live on. Even patients with locked‐in syndrome (LiS), who have lost almost all their mobility and verbal communication, vary in their assessment of their quality of life and well‐being, depending on the specific circumstances and their individual ideas of a good life [25, 26]. Despite the drastic restrictions imposed by LiS, it cannot be generalised that dying would be better than to continue living for these patients.

As a conclusion, considerations of well‐being in the context of assisted dying should always involve both: an orientation towards objective well‐being goods and an evaluation and contextualisation of these goods in relation to the individual, respecting the subject‐dependence of well‐being. While it is possible to determine the relative weight of beneficence‐based arguments for options to live on based on observable aspects of an individual's well‐being, it is impossible to determine the ‘tipping point’ when death becomes preferable to life, because this depends on the individual's *subjective* evaluation of her quality of life. Based on these conceptual considerations, we will assess in the following section whether beneficence‐based arguments can be used as gatekeeping criteria for assisted dying. Thereafter, we will explore which role beneficence‐based obligations can and should play in assisted dying (Section 4)—and therefore also in clinical ethics consultation concerning assisted dying (Sections 5).

## 3 Beneficence as a Gatekeeping Criterion in Assisted Dying?

Several countries link the permissibility of assisted dying to criteria that cannot be justified by referring to the person's autonomy, but only by beneficence‐based arguments. These criteria include, in particular, the— necessary, but not sufficient—conditions that the requesting person must have a serious, incurable illness, in some US states with a limited remaining life expectancy (e.g., less than 6 months) and unbearable physical or mental suffering which cannot be alleviated [27]. Apparently, these criteria are based on assumptions on when assisted dying is compatible with the well‐being of the requesting person, for example, when her suffering from an incurable disease cannot be alleviated. These assumptions are basedimplicitly—on a judgement from an external (!) perspective under which conditions dying is preferrable to continue living. As we have argued above, we do not have a uniform, generally accepted standard for this tipping point from life to death in pluralistic societies. It therefore seems inappropriate to define general well‐being‐based standards for ethically acceptable requests for assisted dying. The Federal Constitutional Court of Germany, for example, unequivocally emphasized in its ruling of February 26, 2020 that the right to self‐determined dying is not limited to certain situations defined by others: ‘Where an individual decides to end their own life, having reached this decision based on how they personally define quality of life and a meaningful existence, their decision must, in principle, be respected by state and society as an act of autonomous self‐determination’ [28].

Furthermore, the restriction to serious, incurable illnesses would also contradict the established ethical standards in treatment decisions: Patients have the right to refuse life‐sustaining interventions resulting in their death not only in cases of severe, incurable illness, but also if they could be saved with comparatively simple measures and with a good prognosis (cf. the refusal of blood transfusions by Jehovah's Witnesses). In these cases, beneficence‐based obligations to save patients' life have to give way to respecting their autonomy.

In addition, criteria such as ‘*unbearable* suffering’ can hardly be reliably objectified in individual cases: Like in treatment decisions, the individual herself must decide based on her own standards whether or not a state of suffering is bearable for her [29]. In conclusion, beneficence‐based arguments are neither suitable nor justified to serve as gatekeeping criteria for the access to assisted dying. We will argue in the following that beneficence‐based obligations nevertheless can and should play an important role in assisted dying, in the best interest of the requesting person, thereby not undermining or restricting, but strengthening her autonomy.

## 4 Beneficence‐Based Obligations in Assisted Dying Beyond Gatekeeping

### 4.1 Providing High Standards of Care

The principle of beneficence generally requires the promotion of well‐being. In requests for assisted dying, it requires first of all to offer appropriate psychosocial support to the person who wants to end her life and often is in an existential crisis. If the request for assisted dying occurs within the context of a severe illness, beneficence‐based obligations also require offering the person alternative (clinical) management strategies to assisted dying, especially intensified palliative care to relieve suffering or improved support for physical impairments. Furthermore, there are also beneficence‐based obligations towards third parties, for example, family members and close friends of the requesting person who should also be offered appropriate psychosocial support. Professional standards of care in assisted dying therefore should not only include safeguards for autonomous decision‐making, but also provisions of appropriate care and support for the persons and her families.

### 4.2 Supporting Autonomous Decision Making

Furthermore, beneficence‐based considerations can have an important *instrumental* value for promoting the requesting person's autonomy. According to the principle of beneficence, HCPs are obliged to identify and offer those options that, from a professional perspective, best promote the well‐being of the persons concerned. This is one of the central tasks in a *shared decision‐making* (SDM) process in clinical medicine—and it seems ethically justified based on a more sophisticated concept of autonomy to also apply this standard to the conversations with persons who consider assisted dying. Possible alternatives to assisted dying must be identified from a beneficence‐based perspective, each with an explicit justification of the extent to which they can have positive effects on the requesting person's well‐being—and in particular on her quality of life. In accordance with the ideal of a deliberative model of the physician‐patient relationship [30], it seems appropriate to respectfully challenge the person with a clear beneficence‐based judgement about which course(s) of action would be in her best interest. This enables the person to critically reflect her wish for assisted dying in the light of alternative courses of action. Ideally, this can further strengthen her autonomy: if she decides in favour of assisted dying after seriously considering possible alternatives, her decision fulfils the criteria of a well‐reflected, autonomous decision—and not just minimum requirements of autonomy. The beneficence‐based obligations thereby serve the person's autonomy. It must be emphasized that at the end of the SDM process, it is the person herself who has full discretion to make her very individual decision. Furthermore, this common deliberation can make it easier for the assisting persons to comply with the person's wish for assisted dying if they have witnessed how thoroughly and seriously she has considered possible alternatives and their advantages.

An interesting question is whether there may be situations in which it is ethically justifiable or even mandated for HCPs to proactively suggest assisted dying as a further possible ‘management strategy’, for example, if a patient is in a state of severe physical and/or mental suffering and has not yet considered this option herself. If assisted dying is accepted as a legitimate practice in a given society, proposing assisted dying to a patient as a further option must be considered as ethically legitimate—even if assisted dying cannot be inferred from the traditional goals of medicine. This applies in particular to cases in which severe suffering is difficult to alleviate (e.g., dystonic movement disorders) or only by means of palliative sedation. Other cases may include situations in which primary functions are drastically reduced (see Section 2), for example, if abilities to participate in life are extremely restricted due to severe and irreversible physical impairments. This would allow the suffering person to decide, based on her subjective conception of a good life, whether she has reached the tipping point where dying is preferable to continue living. Proactively addressing assisted dying in conversations with patients as a *possible* course of action could therefore be ethically justified in these—presumably rare—cases.

### 4.3 Ethical Guidance for Assisting Persons

Furthermore, beneficence‐based obligations can help persons who have been asked for assistance in dying in their individual decision about whether they shall comply with the request or not. Depending on whether there is a better alternative to assisted dying from a beneficence‐based perspective, the person can make an ethically justified decision for herself about complying with the wish for assisted dying or not. In countries where physician‐assisted dying is legal HCPs who are confronted with requests for assisted suicide have the right to decide whether they want to perform the assistance or refrain from doing so, sometimes referred to as ‘conscientious objection’ [31, 32] (for an overview of different legislations on physician‐assisted dying around the world, including regulations regarding the conscientious objection, see [33]). While there are some HCPs who will never provide assisted dying under any circumstances, there are others who are generally willing to do so but want to consider each case individually. For these, the question arises according to which criteria they should decide about providing assistance in dying in each individual case. The fundamental and indispensable ethical prerequisite is that the request is based on a self‐determined, fully responsible decision by the person concerned. If this basic requirement is met, the decision about providing assistance in dying can be based on whether or not there are significantly better alternatives to assisted suicide from the perspective of well‐being: Are key primary functions of the requesting person restricted or no longer existent? Is it likely that they can be improved or restored? Especially: How much restricted are the person's abilities to communicate and participate socially? Even if it is neither possible nor justified—at least in the majority of cases—to determine from the outside when it is better for another person to die than to live on, these considerations can support ethical self‐reflection on whether one would be willing to provide assisted suicide in an individual case. For example, consider a patient with incurable nasopharyngeal carcinoma who requests assisted suicide. Her life‐expectancy is limited to several months and her quality of life is severely compromised. While her pain is controlled with palliative care, there are some symptoms that cannot be controlled (e.g., further paralysis of the cranial nerves, ulceration of the tumour). From the perspective of well‐being, there does not seem to be a clearly better alternative available to the patient. These beneficence‐based considerations may help HCPs who are generally willing to participate in assisted dying but are unsure whether it aligns with their beneficence obligations towards the patient, enabling them to reach a decision in individual cases. However, it must be emphasised once more that it is ethically legitimate to provide assisted suicide even if there are good alternatives from an external well‐being perspective, because of the value we attach to respecting the autonomy of competent persons. For this reason, no *general* criteria for the permissibility of assisted suicide can be derived from this line of argument.

## 5 Structured Ethical Decision‐Making and Ethics Consultation in Requests for Assisted Dying

### 5.1 Principle‐Based Case Discussion as a Method for Structured Ethical Decision‐Making

As in clinical patient care, obligations of beneficence come into play with other ethical obligations, especially respect for the requesting person's autonomy. To further specify the role of beneficence‐based obligations in assisted dying, we therefore would like to show how the relevant ethical obligations can be integrated into a structured ethical work‐up of the individual case. The resulting structure can also be used as guidance for ethics consultations in requests for assisted dying, ideally conducted as a moderated case discussion within the multi‐professional team [34, 35]. This approach involves a step‐wise work‐up of the case, based on a thorough analysis of the *medical and psychosocial situation* of the person and the available (clinical) *management strategies*. We call this approach principle‐based ethical case discussion, because the relevant ethical obligations are determined by the four principles of biomedical ethics: beneficence, nonmaleficence, respect for autonomy, and justice. In a systematic, stepwise workup, the ethical evaluation of the available management strategies should start with the *beneficence‐based obligations*: Which management strategy is in the best interest of the patient regarding her well‐being from a professional perspective? Subsequently, the *autonomy‐based obligations* are evaluated: Which management strategy does the patient prefer after appropriate disclosure about the pros and cons of the available management strategies? The principle of justice finally requires assessing whether there are relevant *obligations to third parties*: Which legitimate interests of other persons affected by the decision, for example, family members, other patients, and the team, must be considered? In the *synthesis*, the elaborated, principle‐based obligations are integrated to an overall ethical evaluation of the case: Do the obligations converge or diverge? If the obligations converge, there are good ethical reasons to perform the respective management strategy. In cases of conflict, the conflicting obligations must be balanced by elaborating good case‐based reasons for which obligation should take priority. The last step shall foster critical self‐reflection: Which is the strongest objection against the option favoured in the synthesis? And to learn from the current case for future similar situations: how could the conflict have been prevented? In the following, we will elaborate on how this method of principle‐based case discussion can be applied in ethics consultations to evaluate requests for assisted dying.

### 5.2 Principle‐Based Ethical Case Discussion in Assisted Dying

According to our experience, a structured work‐up of ethical issues in requests for assistance in dying can also follow the structure of the principle‐based case discussion because the same ethical obligations apply: In addition to promoting and respecting the person's autonomy, we also have beneficence‐based obligations, that is, a concern for the well‐being of the requesting person (see Section 2), and obligations towards third parties.

#### 5.2.1 Overview: Principle‐Based Ethical Case Discussion in Requests for Assisted Dying [cf 34, 35]

1. **Analysis**: *Descriptive* workup of the case
  a. *Psychosocial* and *medical* (if applicable) *situation* of the requesting person
  b. *Available options*, each with the expected further course of life (‘prognosis’), including assisted dying and—if applicable—possible clinical management strategies
2. **Evaluation I**: Ethical obligations towards the requesting person
  a. *Autonomy‐based obligations*: Is the person's request well‐informed with full decision‐making capacity?
  b. *Beneficence‐based obligations*: What are the implications of the available options for the well‐being of the requesting person?
3. **Evaluation II**: *Ethical obligations towards third parties*
  What legitimate interests of other persons involved (esp. family members, team members) should be taken into account?
4. **Synthesis**: *Overall ethical evaluation of the case*
5. **Key question:** Are there *strong beneficence‐based arguments* for the alternatives to assisted dying? What shall be the next steps to put the result into practice?

In the *descriptive work‐up*, the current psychosocial and—if applicable—medical situation of the requesting person and the available options must be elaborated. If the request for assisted dying has developed in the context of severe illness, available clinical management strategies should be discussed in addition to assisted dying: especially optimized palliative care including psychosocial support and forgoing life‐sustaining interventions in the event of life‐threatening crises (e.g., pneumonia). For all options, the likely further course of life should be elaborated (prognosis), regarding length of survival and the expected quality of life, as an essential prerequisite for the following ethical evaluation.

In clinical care and clinical ethics consultations, we usually discuss the beneficence‐based obligations after the descriptive (medical) work‐up. As the central ethical justification for assisted dying is based on respecting the persons autonomy (rather than professional considerations of beneficence), we propose to start the ethical evaluation with the *autonomy‐based obligations* (cf. overview). This requires, first of all, to determine whether the person has full decision‐making capacity or whether it is limited by a mental disorder. A mental disorder in itself, however, does not preclude a fully autonomous decision about assisted dying [29, 36]. In addition, it must be assessed whether the requesting person has been informed about all relevant aspects of the decision and is able to understand and appropriately process this information. Furthermore, it should be a stable decision that is not unduly influenced by external pressure.

Subsequently, the available options must be evaluated according to the *beneficence‐based obligations*, that is, regarding the well‐being of the requesting person. While it is possible to determine how much the options to live on contribute to the person's well‐being, as we have elaborated in Section 2, it is impossible to clearly define the ‘tipping point’ when dying becomes better than to continue living for the requesting person. We therefore propose to limit this step to elaborating on *how strong* the beneficence‐based arguments are in favour of the options available for continuing life. These arguments are weaker the shorter the remaining lifetime is (e.g., in the case of advanced cancer) and the more burdensome and difficult to treat the symptoms are (e.g., pain caused by a dystonic movement disorder).

In assessing the *obligations towards third parties*, the needs of family members or close friends should be taken into account. These persons should also be provided appropriate care and psychosocial support. The interests of assisting persons (e.g., HCPs) can also be discussed in this step of the workup.

In the *synthesis*, we integrate the elaborated ethical obligations into an overall evaluation. In requests for assisted suicide, we propose to specify the key question of this step as follows: Are there strong beneficence‐based arguments for the alternatives to assisted dying? To answer this question, we refer to the results of the second evaluation step, that is, the beneficence‐based obligations. If there are no strong arguments for the options of continuing life based on an assessment of the person's well‐being (e.g., if the remaining lifetime is short and/or the burdensome symptoms are difficult to alleviate), complying with the person's request for assistance in dying is also compatible with the principle of beneficence. This can be ethically reassuring for those who consider assisting the requesting person in terminating her life. However, if there are stronger arguments in favour of the options for continuing life, these arguments should be thoroughly discussed once more with the requesting person to ensure that she has understood and seriously considered the well‐being‐based arguments in favour of continuing to live. If the person remains decided to terminate her life, the assisting persons have a good ethical foundation to comply with the request because they have been reassured that it is the person's well informed and seriously considered decision that matches high standards of autonomous decision‐making. As already indicated above, the persons involved can also use (strong) beneficence‐based arguments to make their own *individual* decision as to whether they are willing to assist the person in dying. At this point, we have to emphasize once again that the beneficence‐based arguments do not question the ethical legitimacy of assisted dying, but offer ethical orientation to *individual* (!) persons in their *personal* (!) decisions as to whether or not they shall assist in dying in the *present* (!) case. If the case is discussed in the interdisciplinary, multiprofessional team, the presence of the various team members can finally be used to discuss and plan the next steps for putting the elaborated result into practice.

## 6 Conclusions

In this paper, we have elaborated the role of beneficence‐based obligations in requests for assisted dying and suggested an integrated structured ethical workup for ethical decision‐making in assisted dying that can be applied as guidance in clinical ethics consultations. Contrary to what some assisted dying laws suggest, beneficence cannot serve as a gatekeeping criterion in regulating access to assisted dying. Legislations determining the legitimacy of assisted dying based on the well‐being of the requesting persons, such as a severe, incurable disease and irremediable suffering, is implicitly based on a judgement (from an external perspective) about when life is so bad that death seems preferable for the person. However, there are no universal standards for these judgements, as we have shown in our brief philosophical analysis of the concept of well‐being, the focus of beneficence‐based obligations. Accordingly, a hybrid understanding of well‐being is most appropriate also in the context of assisted dying: On the one hand, certain objective indicators can be identified which allow to recognise from an external perspective that a person's well‐being may be significantly impaired, in particular in cases of restricted primary functions (including social interaction), high symptom burden, and a short life expectancy. On the other hand, the critical ‘tipping point’ at which the restrictions and burdens are so great that dying becomes preferrable to continuing to live on, can only be determined from the individual's subjective perspective on her own well‐being and quality of life, depending on her individual conception of a good life and the specific circumstances. Along the lines of these conceptual considerations of well‐being, we explored three ways in which the principle of beneficence can provide ethical guidance in dealing with persons requesting assisted dying: (1) It requires high standards of care by offering appropriate psychosocial support for the person and her family, and alternative (clinical) management strategies where available. (2) By explicitly including professional considerations of well‐being in the shared decision‐making process, it can support the requesting person in making a well‐considered, autonomous decision. (3) Finally, it can provide ethical guidance for those who consider to provide assistance in dying in their individual deliberation about whether they personally consider it ethically acceptable for themselves to comply with the request in a specific case.

The principle of beneficence requires reflection on whether there are alternative courses of action that could contribute to the well‐being of the requesting person, for example, by improving important primary functions such as social participation or relieving burdensome symptoms. This provides guidance on how strong the arguments related to the person's well‐being are in favour of alternatives to assisted dying. However, the reflection can also reveal that dying may be an option worth considering also from a professional beneficence perspective, especially if suffering is difficult to alleviate by palliative care or only with the use of palliative sedation, or if primary functions are severely impaired and cannot be restored. In such situations, proposing assisted dying as a further option can be regarded as ethically legitimate.

We suggested a stepwise, principle‐based approach to illustrate how the beneficence‐based obligations can be integrated with other relevant ethical obligations into ethical decision‐making in assisted dying. It can provide guidance for ethics consultations in cases where there is uncertainty among the (potentially) assisting persons about the ethical acceptability of complying with a request for assisted dying: the more sophisticated understanding of well‐being can help to specify beneficence‐based obligations in assisted dying and thereby contribute to decisions with a more comprehensive ethical justification. Overall, a greater focus on beneficence‐based obligations may not only improve the care for persons who wish to die and for their families but also contribute to ethically sound decisions on behalf of the persons considering to assist in dying.

## Conflicts of Interest

Georg Marckmann is a trainer for clinical ethics consultation services and has received financial compensations for the trainings from several German hospitals. Anna Hirsch has no conflict of interest to declare.
